# Mep72, a Metzincin Protease That Is Preferentially Secreted by Biofilms of Pseudomonas aeruginosa

**DOI:** 10.1128/JB.02404-14

**Published:** 2015-01-21

**Authors:** Ian J. Passmore, Kahoko Nishikawa, Kathryn S. Lilley, Steven D. Bowden, Jade C. S. Chung, Martin Welch

**Affiliations:** a Department of Biochemistry, University of Cambridge, Cambridge, United Kingdom; b Department of Traumatology and Critical Care Medicine, National Defense Medical College, Saitama, Japan

## Abstract

In this work, we compared the profile of proteins secreted by planktonic and biofilm cultures of Pseudomonas aeruginosa using two-dimensional difference gel electrophoresis (2D-DiGE). This revealed that a novel metzincin protease, Mep72, was secreted during biofilm growth. Subsequent Western blotting and reverse transcription-PCR (RT-PCR) analyses demonstrated that Mep72 was expressed only during biofilm growth. Mep72 has a tridomain structure comprised of a metzincin protease-like domain and two tandem carbohydrate-binding domains. Unlike the only other metzincin (alkaline protease; AprA) in P. aeruginosa, Mep72 is secreted through the type II pathway and undergoes processing during export. During this processing, the metzincin domain is liberated from the carbohydrate-binding domains. This processing may be self-catalyzed, since purified Mep72 autodegraded *in vitro*. This autodegradation was retarded in the presence of alginate (an extracellular matrix component of many P. aeruginosa biofilms). The expression of full-length *mep72* in Escherichia coli was toxic. However, this toxicity could be alleviated by coexpression of *mep72* with the adjacent gene, *bamI*. Mep72 and BamI were found to form a protein-protein complex *in vitro*. 2D-DiGE revealed that the electrophoretic mobility of several discrete protein spots was altered in the biofilm secretome of an *mep72* mutant, including type III secretion proteins (PopD, PcrV, and ExoS) and a flagellum-associated protein (FliD). Mep72 was found to bind directly to ExoS and PcrV and to affect the processing of these proteins in the biofilm secretome. We conclude that Mep72 is a secreted biofilm-specific regulator that affects the processing of a very specific subset of virulence factors.

## INTRODUCTION

*Pseudomonas aeruginosa* is an opportunistic human pathogen and a major cause of chronic infections in individuals with cystic fibrosis (CF). Chronic P. aeruginosa infections have long been associated with a biofilm mode of growth, characterized by the formation of sessile microbial communities and the production of exopolysaccharide (1–4). Such infections are particularly difficult to eliminate due to reduced immune clearance and their high tolerance to antibiotic treatment (5, 6). However, the biofilm phenotype remains poorly defined. A number of proteomic and transcriptomic analyses have been employed to investigate the lifestyle changes associated with the transition from a planktonic growth mode to a biofilm growth mode (7–12). In spite of this, there remains little consensus on what defines a biofilm (13).

P. aeruginosa is also a secretor. Secreted virulence factors are partly responsible for causing the extensive tissue damage associated with P. aeruginosa acute infections (14). Proteinaceous virulence factors are exported through the numerous secretion systems encoded by the P. aeruginosa genome (15). Many of these secreted proteins are hydrolytic enzymes which include the type I-secreted alkaline protease, AprA, and various type II-secreted proteases, such as elastase (LasB), staphylolysin (LasA), and PvdS-regulated protease (PrpL). Other virulence determinants are secreted through the type III secretion system (T3SS). The T3SS has been proposed to inject effector proteins directly into the host cell (16, 17). The expression of the T3SS often correlates with severe disease and increased mortality (18, 19).

A pair of two-component sensor systems have been identified that reciprocally regulate T3SS expression and biofilm formation (20, 21). As a result, biofilm cells often are thought of as being less virulent than planktonic cells, and the formation of biofilms is thought to represent a commitment toward chronic infection (22). However, biofilms also have been associated with acute infections (23), and chronic infections do not necessarily involve biofilm formation (24). Moreover, the expression of T3S proteins has been detected in biofilms grown under certain conditions, suggesting that biofilm formation and T3S are not always necessarily mutually exclusive phenotypes (11, 25).

To the best of our knowledge, only a few studies have investigated whether specific secreted proteins are associated with the biofilm growth mode. To date, most previous studies have focused on the production of secreted proteins by planktonic cultures (26–29). However, one study by Toyofuku and colleagues examined the secreted proteins that associated with the extracellular polysaccharide matrix. These workers demonstrated that outer membrane vesicles commonly associate with the biofilm matrix, implying that these vesicles are core constituents of P. aeruginosa biofilms (30). Another study showed that three extracellular proteases (AprA, LasB, and PrpL) were upregulated by Ca^2+^ in a mucoid P. aeruginosa strain (FRD1) grown under continuous-flow conditions (31). These proteases were upregulated concomitantly with the alginate biosynthetic gene, *algD*, in the presence of high levels of Ca^2+^ in the culture medium. A study by Purschke and colleagues compared the secretomes of P. aeruginosa and Candida albicans in mixed biofilms (32). Here, they demonstrated that the diversity of P. aeruginosa proteins secreted in mixed-culture conditions was lower than in single-culture conditions. However, some secreted proteins (such as the virulence factor ToxA and hemophore HasAp) were uniquely expressed in mixed biofilms but were not detected in monocultures. Furthermore, proteinaceous factors, such as the adhesin CdrA, have been shown to be upregulated under biofilm conditions (33). CdrA is thought to contribute to biofilm structural integrity by either cross-linking Psl polysaccharide and/or by tethering Psl to cells.

Recently, Balyimez et al. reported that the transcription of two uncharacterized P. aeruginosa open reading frames (ORFs), PA2782 and PA2783, is under the control of the cyclic AMP (cAMP)-responsive transcriptional regulator, Vfr, and that Escherichia coli cells expressing PA2783 displayed proteolytic activity (34). They subsequently renamed PA2783 as Mep72, after the family of metalloendopeptidases to which the protein belongs. In this work, we show that Mep72 is in fact a biofilm-associated secreted protein. We also show that Mep72 binds to the product of its coregulated adjacent gene, PA2782, and that this interaction reduces the toxicity of Mep72 when expressed in E. coli cells. We also demonstrate that Mep72 autocatalytically degrades *in vitro* and is processed *in vivo*. Finally, we demonstrate that Mep72 expression in biofilms specifically affects proteins exported by the type III secretion machinery.

## MATERIALS AND METHODS

### Strains and growth conditions.

Bacterial strains and plasmids used in this study are listed in Table S1 in the supplemental material. P. aeruginosa cultures were grown at 37°C in AGSY medium (56 mM alanine, 17 mM K_2_HPO_4_, 86 mM NaCl, 100 μM CaCl_2_, 10 mM MgSO_4_, 5 μM FeCl_2_, 7.5 μM ZnCl_2_, 0.5% [vol/vol] glycerol, 3 g/liter yeast extract, pH 7). P. aeruginosa forms robust biofilms in AGSY medium, and transcriptomic/proteomic data are available (11, 35). Planktonic cultures were grown in flasks with vigorous shaking and harvested after 3 h (late exponential phase; optical density at 600 nm [OD_600_], ∼1) or 9 h (stationary phase; OD_600_, ∼9). Continuous-flow biofilms were grown in AGSY medium as previously described by Mikkelsen et al. (35).

E. coli cells were grown in Luria broth (LB) or on LB agar (1.5% [wt/vol] Bacto agar) plates.

Carbenicillin was used at final concentrations of 200 μg ml^−1^ for P. aeruginosa and 50 μg ml^−1^ for E. coli. Isopropyl-β-d-thiogalactopyranoside (IPTG) was used at a final concentration of 0.3 mM for the induction of protein expression.

### Plasmid construction.

PAO1 genomic DNA was used as a template for PCR-based amplification of the *bamI* (for biofilm-associated metzincin inhibitor and *mep72* ORFs. The primers are listed in Table S2 in the supplemental material. The maltose-binding protein (MBP)–Mep72 expression plasmid [pMal-P2X(*mep72*)] was constructed by introducing the PCR-amplified *mep72* ORF (minus the first 20 amino acid residues, which encode the endogenous signal sequence) into the EcoRI and HindIII sites of pMal-P2X. The BamI-MBP-Mep72 expression plasmid [pMal-P2X(*bamI*, *mep72*)] was constructed by introducing the PCR-amplified *bamI* ORF into the NdeI site of pMal-P2X(*mep72*). The His_6_-BamI expression plasmid [pQE80(*bamI*)] was constructed by introducing the PCR-amplified *bamI* ORF into the BamHI and HindIII sites of pQE80. The metzincin domain and carbohydrate-binding domain truncates [pMal-P2X(*mep72*-Met) and pMal-P2X(*mep72*-CBM)] were constructed by introducing the respective PCR-amplified domains of the *mep72* ORF into the EcoRI and HindIII sites of pMal-P2X. All cloned inserts were verified by sequencing.

### Protein extraction.

Planktonic cell cultures were sedimented by centrifugation (3,200 × *g*, 30 min, 4°C). Biofilm effluent was collected on ice, and planktonic cells were removed by centrifugation. The average number of planktonic cells in the effluents before clarification was 2 × 10^7^ CFU/ml (in comparison, planktonic cultures in stationary phase had an average cell density of 2 × 10^10^ CFU/ml). The mean protein concentration in the 1-day biofilm effluents was 0.94 ± 0.30 μg/ml. In comparison, the planktonic stationary-phase (PS) samples contained 2.32 ± 0.79 μg/ml protein. Supernatants from the biofilm and planktonic cultures were passed through a 0.2-μm filter (Millipore), and protein was precipitated by the addition of solid trichloroacetic acid to a final concentration of 12.5% (wt/vol). Protein was left to precipitate overnight at 4°C and was collected by sedimentation (3,200 × *g*, 30 min, 4°C). Pellets were washed three times in 50% ethanol and redissolved in 3-[(3-cholamidopropyl)-dimethylammonio]-1-propanesulfonate (CHAPS) buffer (6 M urea, 2 M thiourea, 10 mM Tris, 4% CHAPS detergent, pH 8.5). Intracellular proteins (for Western blotting) were prepared by normalizing cell pellets to the same optical density (OD_600_) using TE buffer (50 mM Tris, 4 mM EDTA, pH 8.3) and sonication on ice. Cell debris was removed by centrifugation (3,200 × *g*, 30 min, 4°C).

### Proteomic analysis.

Samples were harvested and prepared for proteomic analysis essentially as described by Mikkelsen et al. (35), with minor modifications. Briefly, for the growth-mode secretome comparison, four biological replicates were analyzed for each growth condition. Planktonic cells and biofilms of P. aeruginosa PAO1 were grown in AGSY medium as previously described (35). Planktonic cells were harvested in early stationary phase. Biofilms were grown in a continuous flow system (35), and extracellular proteins were harvested for proteomic analysis after 24 h by collecting ca. 150 ml of the tube effluent directly into a flask packed in ice. Culture supernatants were clarified by centrifugation (8,000 × *g*) and filtration (0.2-μm filter; Millipore Stericup). Proteins were precipitated by addition of trichloroacetic acid (10%, wt/vol, final concentration) and harvested by centrifugation (100,000 × *g*, 4°C, 45 min). Pellets were washed with 50% ethanol and resuspended in ASB14 buffer (35). Samples were analyzed by two-dimensional difference gel electrophoresis (2D-DiGE), and statistical analysis was performed using BVA (biological variance analysis) and HCA (hierarchical cluster analysis) as previously described (35). Master numbers were assigned by the DeCyder software and refer to the position of the protein spot on the master gel. For the proteomic comparison of the *mep72* mutant and wild-type secretome, a single biological sample was analyzed using dye-swapped technical replicates, and the modulated protein spots were identified as described above. For the comparison of the *mep72* mutant and wild-type secretome, protein spots were selected for mass spectrometry analysis based on whether they were present in one sample and absent from the other, as opposed to protein spots that were modulated between samples. The samples were analyzed by 2D-DiGE, and statistical analysis was performed using biological variance analysis and hierarchical cluster analysis. Protein spots were excised and digested for liquid chromatography-tandem mass spectrometry (LC-MS/MS) analysis, and the output data were used to search the Pseudomonas aeruginosa database using MASCOT. Protein identifications were considered significant only if the MASCOT score was >80 and they contained one or more peptides with an E value of <0.01. Hits comprising more than one protein identification were discarded from further analysis.

### Western blot analysis.

The protein concentration in samples was quantified spectrophotometrically using Bradford reagent (Sigma). Equal amounts of protein from cell-associated and secreted fractions were separated on 12% sodium dodecyl sulfate-polyacrylamide gels, transferred to polyvinylidene fluoride membranes, and analyzed by Western blotting. ExoS and PcrV primary antibodies were a gift from Arne Rietsch, Case Western Reserve University. Polyclonal anti-Mep72 primary antibody was prepared from rabbits that had been immunized with a peptide corresponding to residues CGIGYMGSGDKNSGR of the metzincin domain (residues 126 to 140) of Mep72. Immunization and antibody preparation was carried out by BioGenes (Germany). Anti-His_6_ primary antibody was purchased from Cell Signaling Technology. Enhanced chemiluminescence peroxidase-labeled anti-rabbit IgG antibodies (Sigma) were used as secondary antibodies. Blots were developed using Immobilon Western chemiluminescent horseradish peroxidase (HRP) substrate (Millipore).

### Protein refolding.

Inclusion bodies of His_6_-Mep72 were harvested and solubilized in 50 mM Tris-HCl (pH 8) containing 300 mM NaCl, 15 mM imidazole, and 8 M urea. The solubilized protein solution was clarified by centrifugation (9,000 × *g*, 30 min, 20°C) and loaded onto a 2-ml (packed bed volume) nickel-nitrilotriacetic acid (Ni-NTA) column equilibrated in the same buffer. The column was loaded and washed at room temperature. The column was washed with ca. 200 ml equilibration buffer. Elution was done by increasing the imidazole concentration to 300 mM. For refolding, the eluted protein was rapidly diluted into a 20-fold volumetric excess of 50 mM Tris-HCl (pH 8) containing 200 mM NaCl. Where indicated, the refolding buffer was supplemented with sugars or polymers.

MBP-tagged Mep72 was purified in a similar way. Inclusion bodies were harvested and solubilized in 8 M urea. The sample then was rapidly diluted by squirting it into a large volumetric excess of rapidly stirred refolding/column buffer (20 mM Tris-HCl [pH 7.4] containing 200 mM NaCl and 1 mM EDTA) at 4°C. After 60 min, the sample was clarified by centrifugation and the soluble supernatant was loaded directly onto an amylose affinity resin. The column was washed overnight, and the bound protein was eluted in column buffer containing 10 mM maltose. Eluted fractions were pooled and concentrated by ultrafiltration (10,000 molecular weight cutoff; Millipore) in a centrifugal concentrator.

### Immunoprecipitation (pulldown).

His_6_-BamI and MBP-Mep72 were expressed in E. coli DH5α from pQE80(*bamI*) and pMal-P2X(*mep72*), respectively, in 1 liter of LB. Cells were grown with vigorous shaking at 37°C to mid-log phase, after which protein expression was induced by the addition of IPTG to a final concentration of 0.3 mM. Cells subsequently were grown at 25°C overnight with shaking. Cells were sedimented by centrifugation (4,000 × *g*, 20 min, 4°C), and the pellets were resuspended in buffer A (50 mM Tris, 200 mM NaCl, pH 8.0). The cells were lysed by sonication on ice. Both recombinant proteins formed insoluble inclusion bodies. The inclusion bodies were collected by sedimentation (9,000 × *g*, 30 min, 4°C) and solubilized in buffer A containing 8 M urea. Insoluble material was removed by centrifugation (9,000 × *g*, 30 min), and the proteins were refolded by rapid injection into a 16-fold volumetric excess of rapidly stirred buffer A. The dilute bait protein suspensions then were loaded onto the appropriate affinity matrix (Ni-NTA–agarose [Qiagen] to capture the His-tagged BamI and amylose resin [New England BioLabs] to capture the MBP-tagged Mep72). The columns then were washed to remove unbound bait protein before applying the target protein solution. (As a control, the target protein solutions also were run through Ni-NTA or amylose columns to which no bait protein had been preadsorbed.) After washing the columns to remove unbound target proteins, the bound proteins were eluted with either 300 mM imidazole (for the Ni-NTA affinity matrix) or 10 mM maltose (for the amylose affinity matrix).

For the ExoS and PcrV pulldown, P. aeruginosa planktonic cultures (eight 25-ml cultures) were grown to stationary phase, and the culture supernatants were passed through a 0.2-μm filter (Millipore). Secreted proteins were concentrated in a 10-kDa cutoff centrifugal concentrator (Millipore) to a final volume of 1 ml (3,200 × *g*, 4°C). The concentrated protein was diluted 20-fold in buffer A before being loaded onto the affinity capture matrix.

### qRT-PCR.

All cells were harvested directly into RNAlater (Ambion), kept at 4°C overnight, and stored at −80°C until use. The culture-RNAlater mixture was thawed on ice, and a suitable amount of cells was sedimented by centrifugation. Cells were resuspended in water containing 1 mg lysozyme ml^−1^ (Sigma) and incubated for 15 min at room temperature. RNA was extracted using an RNeasy Mini purification kit (Qiagen) by following the manufacturer's instructions. The resulting RNA (200 ng) was used as a template for reverse transcription and conversion into cDNA using Superscript II reverse transcriptase (Invitrogen) by following the manufacturer's instructions. Quantitative real-time reverse-transcription PCR (qRT-PCR) was performed on the cDNA using 1× SYBR green PCR master mix (Applied Biosystems) with 10 pmol of the appropriate primers (see Table S2 in the supplemental material). Amplification was carried out using an ABI PRISM 7300 real-time PCR system, and fluorescence data were processed using SDS software (ABI). Relative gene expression was obtained using 16S rRNA as the control, with mRNA/16S rRNA from each sample set equal to 1. Three biological replicates were examined for each growth condition.

## RESULTS

### Biofilm and planktonic secretome comparison by 2D-DiGE.

**FIG 1:**
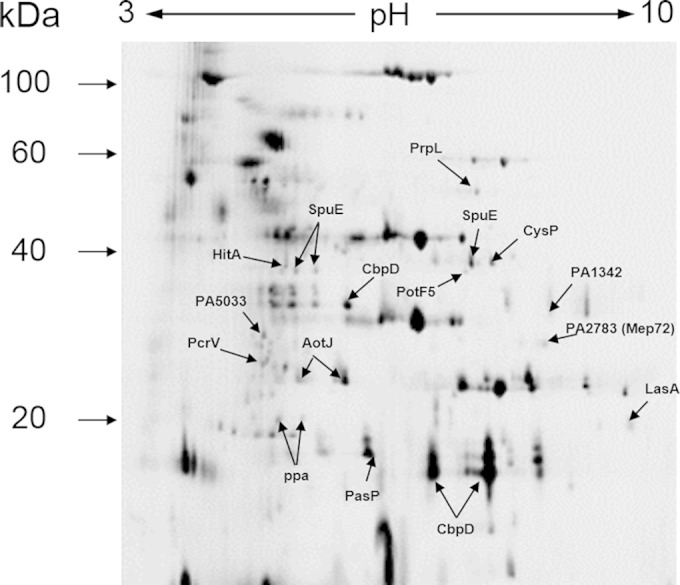
Proteomic analysis of the biofilm and planktonic cell secretome fraction. The fluorescence image of a Cy2-labeled pooled internal standard is shown. A selection of the protein spots that were identified by LC-MS/MS are indicated.

We wanted to test directly whether biofilms of P. aeruginosa secrete an altered spectrum of proteins compared with planktonic cells. Continuous-flow biofilms were grown in silicon tubing as previously described (35), and secreted proteins were harvested. The secretome obtained from PAO1 biofilms grown for 1 day (1dB) was compared with that harvested from a stationary-phase planktonic culture of PAO1 (PS). The secretome samples were analyzed by quantitative 2D-DiGE (Fig. 1). One hundred sixteen spots were modulated in the biofilm samples (93 upregulated, 13 downregulated; *P* ≤ 0.01). A selection of the most highly modulated spots were excised from the gels and identified by LC-MS/MS and MASCOT searching of the P. aeruginosa database (Table 1). Several spots corresponding to known virulence factors were upregulated in the biofilm secretome, including LasA, PasP, and PcrV. Additional upregulated spots corresponded to SpuE, AotJ, PA1342, and PotF5, all of which are predicted to be involved in polyamine uptake. Polyamines have been shown to play a role in the tolerance of P. aeruginosa to some antibiotics (36–38), although the mechanism underlying this currently is unknown. Other upregulated proteins included the ferric iron receptor (HitA; perhaps indicative of a shortage of ferric iron in the biofilm), the sulfate binding protein of the sulfate ABC uptake system (CysP), and a PvdS-regulated protease (PrpL). PvdS is an alternative sigma factor that stimulates the expression of a siderophore (pyoverdine) as well as other genes (e.g., *prpL* [39]), again indicating iron limitation in the biofilm. Finally, we observed several spots corresponding to the chitin-binding domain protein CbpD, previously known as LasD. Two of the CbpD spots were less abundant in the biofilm secretome and one was more abundant. CbpD is known to be processed by other secreted proteases (40), and we suspect that this explains the concomitant appearance of apparently up- and downregulated spots.

**TABLE 1:** Summary of the identified protein spots modulated in the biofilm-grown culture secretome compared with their activity in the planktonic culture secretome

Function and PA no.	Master no.	Gene name	Gene product	Level of abundance^*a*^	Modulated ratio^*b*^ (biofilm/PS)
Transport of small molecules					
PA0301	1022	*spuE*	Polyamine transport protein	↑	19.8
	1033			↑	10.0
	971			↑	13.6
PA4687	996	*hitA*	Ferric iron-binding periplasmic protein HitA	↑	17.5
PA2592	1019	*potF5*	Probable periplasmic spermidine/putrescine-binding protein	↑	33.9
PA1493	960	*cysP*	Sulfate-binding protein of ABC transporter	↑	6.4
PA0888	1469	*aotJ*	Arginine/ornithine binding protein AotJ	↑	17.9
	1480			↑	14.1
PA1342	1155		Probable lysine/arginine/ornithine-binding protein	↑	9.9
Known virulence factors					
PA0423	1765	*pasP*	P. aeruginosa small protease PASP	↑	5.2
PA1871	1687	*lasA*	LasA precursor	↑	16.5
PA4175	663	*prpL*	PvdS-regulated endoprotease, protease IV	↑	39.1
PA1706	1404	*pcrV*	Type III secretion protein PcrV	↑	17.6
PA0852	1170	*cbpD*	Chitin-binding protein CbpD precursor	↑	8.0
	1844			↓	37.9
	1830			↓	85.3
Unclassified hypothetical proteins					
PA5033	1300		Hypothetical protein PA5033	↑	15.5
PA2783	1313	*mep72*	Hypothetical protein PA2783	↑	18.1
Central intermediary metabolism:	1721	*ppa*	Inorganic pyrophosphatase	↓	3.9
PA4031	1713			↓	3.4

a ↑ and ↓ indicate whether the spot was more or less abundant, respectively, in the biofilm secretome than in the planktonic secretome.

b Modulated ratio represents the fold change in the biofilm secretome compared to that in the planktonic secretome.

An uncharacterized protein, PA5033, also was upregulated in the biofilm secretome. PA5033 is predicted to form a bicistronic operon with the adjacent gene, PA5032 (41). PA5032 is predicted to encode an AraC-like transcriptional regulator. The PA5032-PA5033 cluster is found only in P. aeruginosa and not in any other sequenced Pseudomonas spp., suggesting that these genes are specifically important for the lifestyle of P. aeruginosa. Bioinformatic analysis of the PA5033 amino acid sequence revealed that it is comprised of 6 or 7 consecutive blocks of repeated sequence, reminiscent of the β-propeller structure associated with FG-GAP-containing proteins, integrins, and adhesins (42, 43). However, a profile screen (MOTIF, KEGG2) of the repeated unit revealed no significant matches in the Swiss-Prot database.

**FIG 2:**
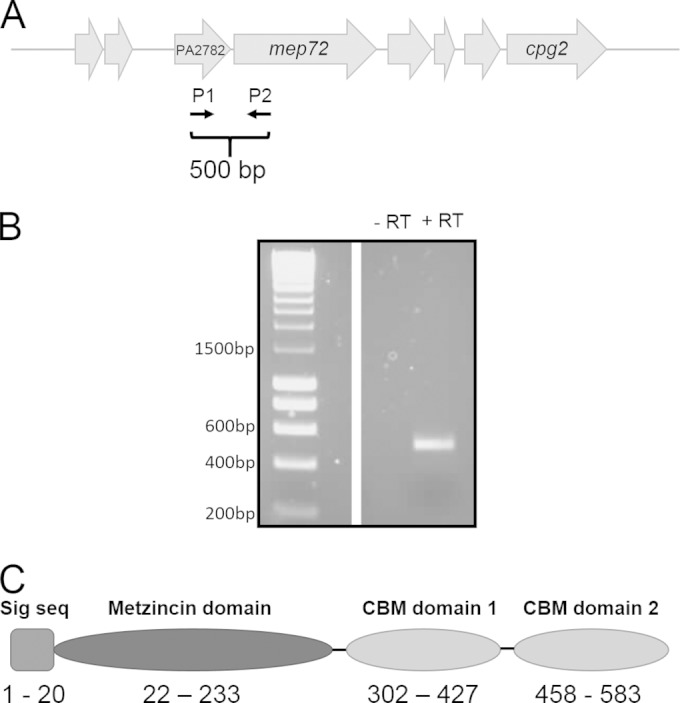
Bioinformatic analyses of PA2783 (Mep72). (A) Genomic context of *bamI* (PA2782) and *mep72*. Schematic locations of the primers used to confirm the operonic organization of *bamI* and *mep72* are indicated. (B) cDNA (+RT) from biofilms was used as a template for PCR with the primers indicated in panel A to amplify the intergenic region between *bamI* and *mep72*. A reaction without reverse transcriptase (−RT) was carried out as a control. (C) Predicted domain structure of the metzincin Mep72. Numbers correspond to the amino acid residues comprising each domain and the predicted secretion signal sequence (Sig seq). The full-length protein is 599 residues long.

We also noted that PA2783 (Mep72) was 18-fold upregulated in the biofilm secretome. *mep72* is predicted to form a bicistronic operon with its adjacent gene, PA2782 (Fig. 2A). To test this, we harvested RNA from PAO1 biofilms. The RNA was converted to cDNA and used as a template to PCR amplify across the junction between PA2782 and *mep72* using primers P1 and P2 (Fig. 2B). If the genes are transcribed as a single contiguous transcript, a 500-bp product would be anticipated, and this is what was observed (Fig. 2B). No 500-bp product was observed in the no-RT control. We obtained no evidence that PA2782 is cotranscribed with the upstream gene (PA2781) or that *mep72* is cotranscribed with the downstream gene (PA2784) (data not shown).

Pfam domain analysis of Mep72 revealed that the protein is comprised of three domains: an N-terminal metalloprotease domain and two tandem C-terminal carbohydrates (Fig. 2C). The amino acid sequence of the N-terminal domain was analyzed using the sequence-structure homology recognition program FUGUE (44). This analysis suggested that Mep72 is structurally related to snake venom metalloproteases (adamalysins) and snake venom endothelial cell vascular apoptosis-inducing protein (VAP-1), which are members of the metzincin superfamily of zinc-dependent metalloproteases (45, 46). Further inspection of the primary structure of Mep72 revealed that it contained both of the conserved sequence motifs (the zinc-binding motif and Met-turn) characteristic of metzincins (46). The bacterial metzincins, also known as serralysins, often are associated with virulence. Indeed, the only other metzincin encoded by the PAO1 genome is the well-established virulence factor AprA (alkaline protease). Unlike AprA, which has a dedicated type I secretion system, Mep72 has an N-terminal signal sequence and is predicted to be targeted to the periplasm via the Sec pathway. Analysis of the amino acid sequence of the C-terminal domains suggests that they are members of the carbohydrate-binding module (CBM) family, usually found in proteins that degrade or bind to polysaccharides (47). The remainder of this study investigates the function and properties of this biofilm-associated metzincin protease. Additionally, we investigate the relationship between *mep72* and its adjacent gene (PA2782), which we designate *bamI* (for biofilm-associated metzincin Inhibitor).

### Expression of Mep72 is upregulated in continuous-flow biofilms.

**FIG 3:**
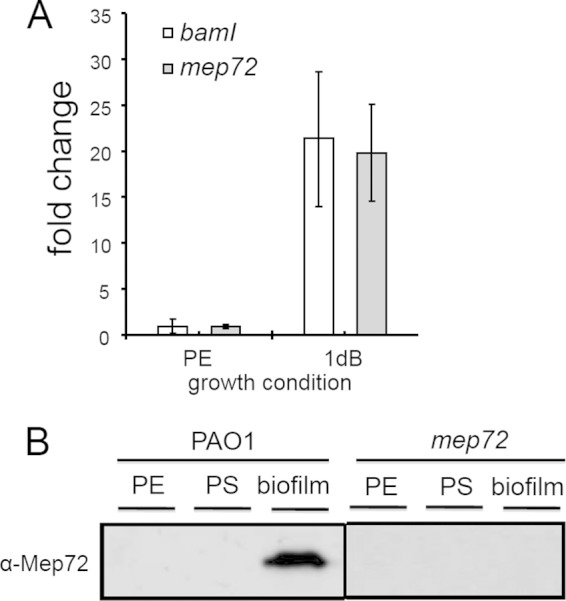
*mep72* expression in biofilms and planktonic cultures. (A) qRT-PCR analysis of *mep72* and *bamI* transcript levels in cells sampled from planktonic exponential-phase (PE) and 1-day-old continuous-flow biofilms (1dB). Similar results were obtained in comparisons of 1dB biofilm-grown cells with planktonic stationary-phase cultures. The error bars represent standard deviations from 3 replicates. (B) Western blot analysis of Mep72 expression and secretion in PE, planktonic stationary-phase (PS), and continuous-flow biofilms of the wild-type (PAO1) and the *mep72* mutant.

In order to determine the transcriptional expression profile of *mep72*, qRT-PCR was carried out on RNA harvested from biofilm cells (1dB) grown in a continuous-flow setup and from cultures of planktonic cells (Fig. 3A shows the data for exponentially growing planktonic cultures [PE]; essentially the same results were obtained for comparisons of stationary-phase planktonic cultures with biofilm cells [data not shown]). *mep72* transcript levels were 18-fold upregulated in 1dB cells compared with the level for planktonic cells, corroborating the observations from the 2D-DiGE analysis. The expression profile of *bamI* also was assessed by qRT-PCR (Fig. 3A). This revealed that the expression of *bamI* follows that of *mep72* and was upregulated in continuous-flow biofilms, and that *bamI* was poorly expressed in planktonic cells. This is consistent with the notion that *bamI* and *mep72* are functionally linked.

To further verify that Mep72 expression is biofilm specific, secreted proteins from biofilm and planktonic cultures were extracted, and the expression of Mep72 was monitored by Western blotting with an antibody raised to the metzincin domain of Mep72 (Fig. 3B). Mep72 was undetectable in the secretome of aerobically grown PE and PS planktonic cultures but was abundant in continuous-flow biofilm supernatants. No Mep72 was detected in the biofilm secretome of an *mep72* mutant (Fig. 3B). These transcriptomic and proteomic analyses indicate that Mep72 is a biofilm-associated gene product under these growth conditions.

### Mep72 is secreted via the type II secretion system and is processed during export.

**FIG 4:**
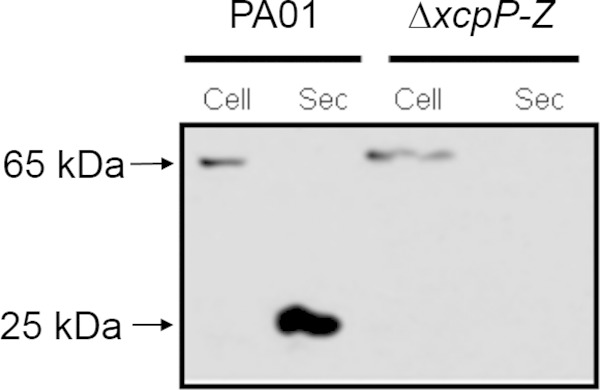
Western blot analysis of Mep72 secretion in biofilms of PAO1 and a Δ*xcpP-Z* mutant. Cell, cell-associated fraction; Sec, secreted fraction.

Since Mep72 is secreted to the extracellular milieu and has a predicted N-terminal Sec secretion signal, it is likely to be exported via the type II general secretory pathway (GSP). Therefore, we predicted that Mep72 would serve as a substrate for the Xcp system, the main terminal branch of the GSP in P. aeruginosa (48). To test this, cell-associated and secreted proteins were harvested from biofilms of PAO1 and an Δ*xcpP-Z* mutant and compared by immunoblot analysis (Fig. 4). Comparison of the cell-associated fractions demonstrates that full-length Mep72 (65 kDa) is expressed in both strains. However, comparison of the secreted fraction reveals that while Mep72 is detectable in the biofilm secretome of PAO1, it is absent from the secretome of an Δ*xcpP-Z* mutant. This suggests that Mep72 is indeed secreted through the Xcp pathway. The data presented in Fig. 4 also show that Mep72 undergoes processing during or following its export. The cell-associated protein product corresponds to the predicted full-length isoform (metzincin domain plus tandem carbohydrate-binding domains) with a molecular mass of 65 kDa. However, the secreted protein product has a lower molecular mass (∼25 kDa) and, given its size and reactivity with the antibody (which was raised against a sequence of amino acids in the metzincin domain), most likely corresponds to the metzincin domain only. The same 25-kDa processed form was observed in the biofilm secretome of mutants containing disruptions in genes encoding the major P. aeruginosa secreted proteases (LasA, LasB, AprA, and PrpL) (data not shown), suggesting that the protein is processed by a minor protease or that it undergoes autoprocessing. Autoprocessing is not uncommon for members of the metzincin protease superfamily. Metzincin proteases often are translated as inactive precursor peptides which subsequently undergo an autoprocessing event that yields their enzymatically active, mature form (46).

### Ectopic expression of Mep72 in E. coli is toxic.

**FIG 5:**
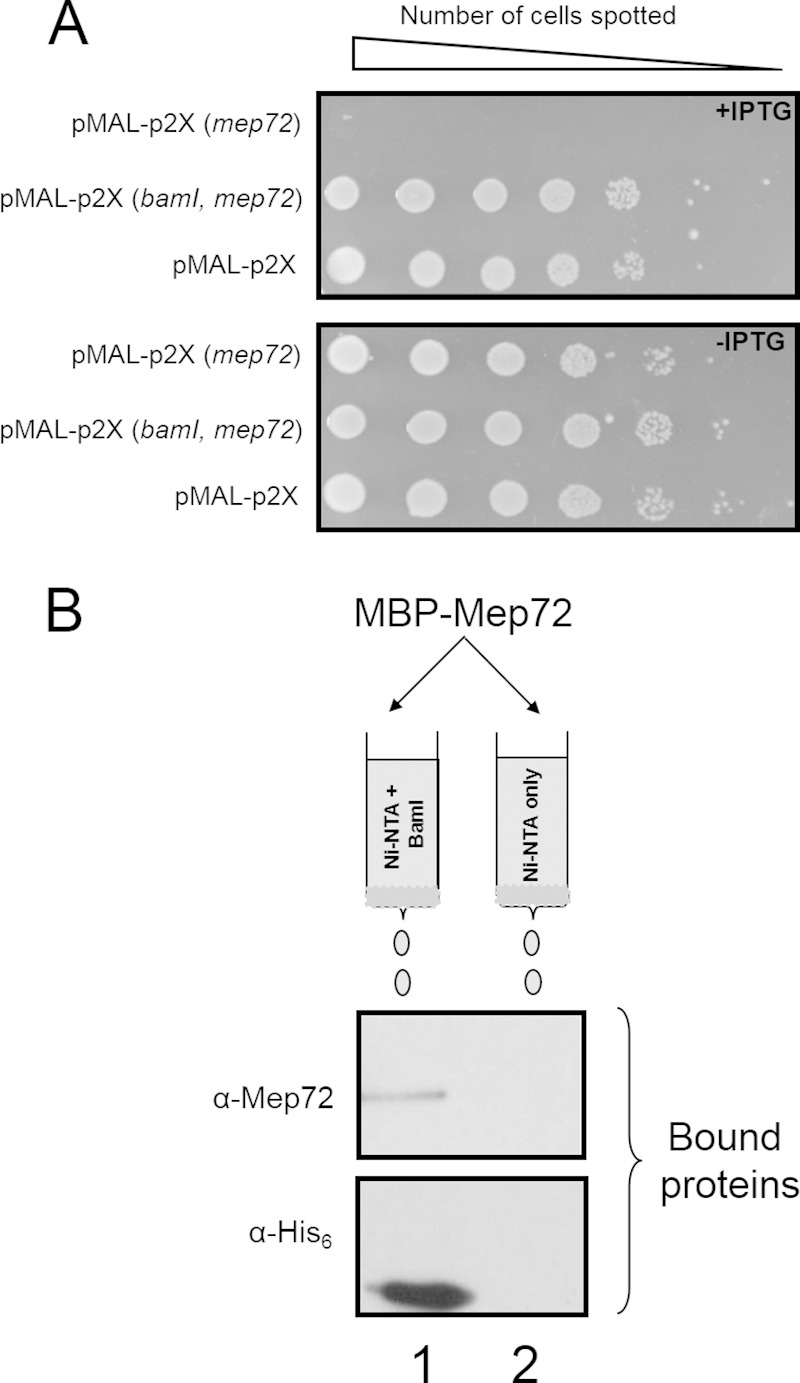
Ectopic expression of *mep72* in E. coli is toxic but is counteracted by coexpression of *mep72* with *bamI*. (A) Serial dilutions of E. coli carrying plasmid-encoded, IPTG-inducible MBP-Mep72 [pMAL-p2X(*mep72*)], BamI-MBP-Mep72 [pMAL-p2X(*bamI*, *mep72*)], and empty pMAL-p2X vector control (pMAL-p2X) were spotted onto LB-agar plates with and without IPTG. The spotted dilutions were allowed to grow for 24 h before being photographed. (B) Mep72 forms an isolable complex with BamI. His_6_-BamI was overexpressed in E. coli and refolded. The refolded protein was loaded onto an Ni-NTA affinity matrix. The matrix was washed to remove any unbound BamI. Following this washing, refolded MBP-Mep72 was passed over the matrix. Excess MBP-Mep72 was washed off, and the bound proteins were eluted in buffer containing 300 mM imidazole. The presence of BamI and Mep72 in the column loadings and eluates was determined by Western blotting using anti-His_6_ antibodies or anti-Mep72 antibodies, as indicated. As a control, we measured the binding of MBP-Mep72 to Ni-NTA beads that had not been pretreated with His_6_-BamI. Lane 1, eluate from column precharged with His_6_-BamI after adding 300 mM imidazole; lane 2, eluate from control column after adding 300 mM imidazole.

To further investigate the properties of Mep72, we recombinantly expressed the gene in E. coli DH5α. An N-terminal maltose-binding protein (MBP) with Sec leader peptide was fused to the mature portion (residues 21 to 599) of the *mep72* ORF and expressed from an IPTG-inducible promoter. Upon induction with IPTG, a decrease in cell viability was observed (Fig. 5A). This reduction in cell viability suggests that recombinant Mep72 expression is toxic to the cell. We speculated that the product of the cotranscribed *bamI* gene functions to counteract this effect. To test this, *bamI* was cloned upstream of the MBP-Mep72 fusion protein. In this construct, both genes (*bamI* and *mep72*) were expressed from the same IPTG-inducible promoter. Upon induction with IPTG, cell viability was restored to the level of the plasmid-only control (Fig. 5A). This suggests that BamI is essential for counteracting the toxic effect(s) of Mep72. This is reminiscent of the situation encountered with the alkaline protease, AprA, and its cognate inhibitor protein, AprI (49–51). Although the exact mechanism of AprI action remains unclear, it has been proposed that AprI binds to AprA, preventing premature protease activity until the protein has been secreted.

### Mep72 and BamI physically interact *in vitro*.

To determine whether the countertoxic effect of BamI on Mep72 was mediated through a physical interaction between the two proteins, a pulldown assay was carried out. Briefly, BamI containing an N-terminal hexahistidine tag (His_6_-BamI) was expressed in E. coli, refolded *in vitro*, and immobilized on an Ni-NTA affinity matrix. In a separate culture, MBP-Mep72 was expressed, refolded *in vitro*, and then passed through either a clean Ni-NTA affinity matrix (control resin) or through the Ni-NTA matrix to which His_6_-BamI had been attached. The columns were washed, following which the bound proteins were eluted and resolved by SDS-PAGE/Western blotting (Fig. 5B). MBP-Mep72 (105 kDa) bound to the column that was precharged with His_6_-BamI but did not bind the Ni-NTA affinity matrix in the absence of His_6_-BamI. This demonstrates that BamI and Mep72 do indeed physically interact *in vitro* and suggests that the countertoxic effect of BamI on Mep72 is mediated through formation of a protein-protein complex. It should be noted that since both the BamI and Mep72 protein preparations were necessarily obtained by refolding *in vitro* (these proteins partition into insoluble inclusion bodies when overexpressed *in vivo*), it is formally possible that only a small fraction of the proteins were in their physiologically active conformation. Therefore, the extent of coprecipitation observed in Fig. 5B is likely to underrepresent the probable interaction *in vivo*.

### Mep72 has autoproteolytic activity.

As with MBP-tagged Mep72, overexpression of His_6_-tagged Mep72 in liquid cultures of E. coli caused the culture to stop growing, and the protein partitioned into insoluble inclusion bodies. These inclusion bodies were harvested, washed, denatured in 8 M urea, and then refolded by rapid dilution to yield the native protein.

**FIG 6:**
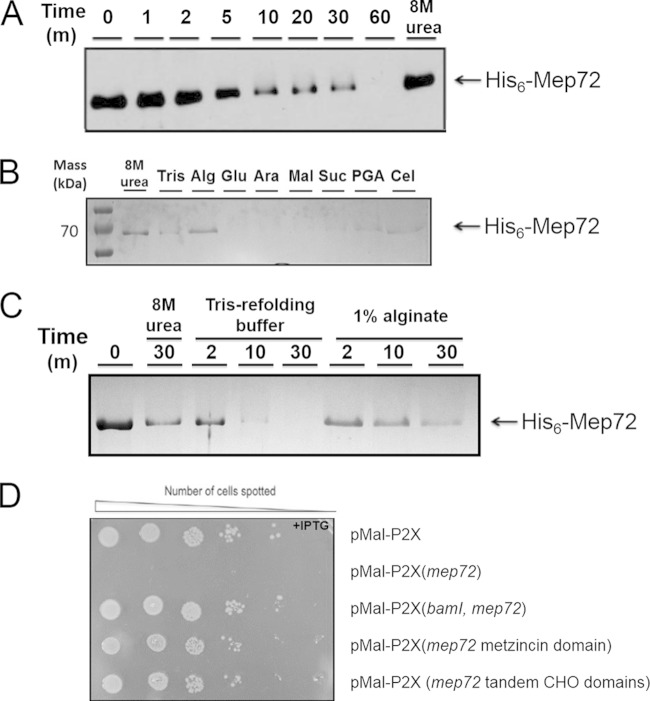
Refolding, autoproteolysis, and toxicity of Mep72. (A) Shown is a Western blot of Mep72 undergoing autoproteolysis. Denatured His_6_-Mep72 was refolded by rapidly diluting the urea denaturant. Samples were incubated for the indicated times before the addition of SDS sample buffer to stop any further reaction. The products were resolved by SDS-PAGE and analyzed by Western blotting. An Mep72 sample incubated for 60 min in 8 M urea (i.e., maintained in a denatured state) was used as a control. (B and C) Alginate retards Mep72 autoproteolysis. (B) Urea-denatured Mep72 was refolded as described for panel A, except that the indicated sugars or sugar polymers were included in the refolding mixture. Reactions were allowed to proceed for 20 min before quenching with SDS sample buffer and resolution by SDS-PAGE. The resulting Coomassie blue-stained gel is shown. The refolding buffers contained Tris-HCl–NaCl alone (Tris) or Tris-HCl–NaCl supplemented with 1% (wt/vol) alginate (Alg), glucose (Glu), arabinose (Ara), sucrose (Suc), maltose (Mal), polygalacturonic acid (PGA), or cellulose (Cel), as indicated. (C) Kinetics of Mep72 autoproteolysis with or without 1% (wt/vol) alginate. His_6_-Mep72 was refolded as outlined above. Aliquots of the refolded protein mixture were removed and quenched after a 2-, 10-, and 30-min reaction, as indicated. Shown is a Coomassie blue-stained gel of the products. (D) The metzincin domain and carbohydrate-binding domains of Mep72 are not themselves intrinsically toxic in the absence of BamI. Serial dilutions of E. coli containing plasmid-encoded, IPTG-inducible N-terminal MBP fusions to the metzincin- and carbohydrate (CHO)-binding domains of Mep72 were spotted onto LB-agar plates containing IPTG. The spotted dilutions were allowed to grow for 24 h before being photographed to record cell viability.

Purified Mep72 did not detectably proteolytically degrade general substrates, such as azocasein, gelatin, or elastin, even after prolonged incubation (data not shown). However, it did undergo rapid autodegradation. As soon as the His_6_-Mep72 was renatured, it began to autodigest (Fig. 6A). Autodigestion was essentially complete after 60 min, with little evidence of partially digested intermediates apparent. In contrast, MBP-tagged Mep72 remained stable for hours, suggesting that the presence of the MBP tag blocks autoproteolysis. To further test this possibility, we treated the MBP-tagged Mep72 with factor Xa, which cleaves at the junction between the two fused proteins. Once again, the liberated Mep72 rapidly autodigested (data not shown). Interestingly, the coliberated MBP tag apparently was unaffected by the released Mep72, again indicating that the observed proteolysis is highly specific. Taken together, these data suggest that recombinant Mep72 is unstable due to autoproteolysis, at least under the conditions prevailing after refolding.

### Mep72 autoproteolysis is retarded by the presence of alginate.

Mep72 contains two C-terminal carbohydrate-binding domains, raising the possibility that Mep72 binds polysaccharides. Therefore, we wondered whether polysaccharides influence Mep72 autoproteolysis. To investigate this, purified His_6_-Mep72 was renatured in the presence of various carbohydrates (including monosaccharides, disaccharides, and polysaccharides), and the subsequent autoproteolysis was monitored by SDS-PAGE (Fig. 6B). Incorporation of alginate into the refolding buffer clearly retarded the rate of autoproteolysis compared with the other sugars tested. These data indicate that the half-life of the protein can be affected by factors that presumably bind to the carbohydrate-binding domains. Further kinetic analyses confirmed that Mep72 autolysis is substantially delayed in the presence of 1% alginate (Fig. 6C).

As shown in Fig. 5A, high-level expression of MBP-tagged Mep72 is toxic to E. coli. However, we noted that prolonged (overnight) incubation of induced liquid cultures of E. coli expressing MBP-tagged *mep72* sometimes led to a restoration in growth, presumably due to the outgrowth of less toxic spontaneous bypass mutants. Western analyses revealed that in most of these mutants, expression of Mep72 was abolished (data not shown). However, in one case (bypass mutant IP28), the *mep72* ORF was found to contain a C→T transition, which led to the introduction of a stop codon in place of Gln_317_. This mutation truncates the protein downstream of the metzincin domain (residues 46 to 175) within the first carbohydrate-binding domain (CBM domain; residues 302 to 427). Western blotting confirmed that IP28 expressed a truncated, metzincin domain-containing protein. The location of this truncation was surprising, since we had assumed that the toxic effects of Mep72 expression in E. coli arose due to inappropriate proteolytic activity mediated by the metzincin domain, yet in IP28 the metzincin domain was intact. Therefore, we examined whether the ectopic expression of the metzincin domain alone or the tandem CBM domains was toxic to E. coli. However, the expression of neither construct was toxic (Fig. 6D). Taken together, these results suggest that the metzincin domain and tandem CBM domains both are required for Mep72 toxicity to be manifest. However, this does not rule out the possibility that the truncation of Mep72 resulted in improper folding, which could account for the observed lack of toxicity.

### Flagella and type III secretion factors are modulated in a *mep72* mutant.

Given the results described above, we surmised that Mep72 is not a general protease and that its target is highly specific. To test whether specific pseudomonal proteins are affected by Mep72 activity, we used 2D-DiGE to compare the proteins secreted by a biofilm of PAO1 with those secreted by a biofilm of the *mep72* mutant. The logic here was that since proteolytic processing often gives rise to a change in the electrophoretic mobility of target proteins on 2D gels, spot shifts between the wild type and *mep72* mutant indicate that the corresponding proteins are targets of Mep72 activity. Consistent with the notion that Mep72 is not a general protease, the secretome of the *mep72* mutant overall was very similar to that of the wild type. However, a small number of discrete changes in spot intensity were clearly observed in the 2D gel electrophoretogram (see Fig. S1 in the supplemental material).

Analysis of the spots more abundant in the PAO1 secretome (Table 2) revealed three isoforms of Mep72, one with a high molecular weight (spot 880) and two more-abundant spots with lower molecular weights (spots 1421 and 1426). The peptide sequences of the two lower-molecular-weight isoforms mapped onto the metzincin domain only, while the peptide sequences of the higher-molecular-weight isoform mapped onto the metzincin domain and the first carbohydrate-binding domain of the protein. These data are consistent with our earlier observation that Mep72 is likely to be processed during or shortly after secretion. Additional protein spots modulated in the PAO1 secretome included the type III secretion translocation factor PcrV and the flagellum-capping protein FliD.

**TABLE 2:** Proteins modulated in PAO1 and *mep72* secretome^*a*^

Function and PA no.	Master no.	Gene name	Gene product	Level of abundance^*b*^	Fold modulation
Spots displaying increased abundance in the wild-type (PAO1) secretome					
PA2783	1426	*mep72*	Metzincin protease Mep72	↑	55.8
PA2783	1421	*mep72*	Metzincin protease Mep72	↑	21.6
PA1706	1483	*pcrV*	Type III secretion protein PcrV	↑	7.9
PA1094	810	*fliD*	Flagella capping protein FliD	↑	7.6
PA2783	880	*mep72*	Metzincin protease Mep72	↑	7.2
Spots displaying decreased abundance in the wild-type (PAO1) secretome					
PA1709	1401	*popD*	Type III secretion protein PopD	↓	7.1
PA3841	757	*exoS*	Type III secretion effector ExoS	↓	5.1
PA3841/PA0044	751	*exoS-exoT*	Type III secretion effector ExoS/ExoT	↓	3.8

a Summary of the identified protein spots modulated in the PAO1 secretome compared to their activity in the *mep72* mutant secretome. While LC-MS/MS analysis identified spot 757 as ExoS, spot 751 was identified as either ExoS or ExoT. Given that ExoS and ExoT share 74% amino acid sequence homology, many of the peptide sequences retrieved following trypsin digestion are almost identical. In these circumstances, ExoS and ExoT cannot be distinguished from each other.

b ↑ and ↓ arrows indicate whether the spot was more or less abundant in the PAO1 secretome than in the *mep72* mutant secretome.

Analysis of the protein spots that were more abundant in the *mep72* mutant secretome revealed additional type III secretion factors. These included the T3SS translocation pore component PopD and the effector proteins ExoS and, possibly, also ExoT (ExoS and ExoT share very high levels of amino acid sequence similarity). These data suggest that Mep72 targets certain secreted T3S and flagellar proteins. Therefore, we postulated that there is a direct physical interaction between Mep72 and the T3SS proteins (see below).

**FIG 7:**
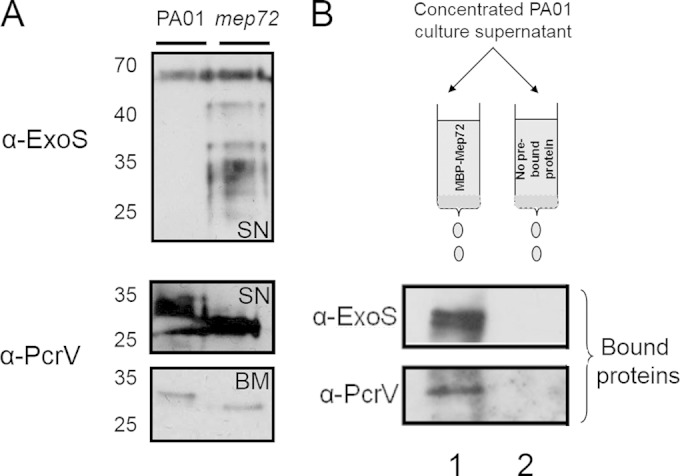
Mep72 binds to T3S factors and prevents proteolytic processing. (A) Western blot analysis of ExoS and PcrV secreted from 1-day-old PAO1 and *mep72* mutant biofilms. The ExoS-containing sample was concentrated culture supernatant (SN). The PcrV-containing samples are from culture supernatant (SN) and cell-free biofilm matrix (BM), as indicated. Note the clear difference in size of PcrV between the wild-type and *mep72* mutant in both sample types. (B) Western blot analysis of pulldown between Mep72 and T3S factors ExoS and PcrV. Planktonic PAO1 culture supernatants (PAO1 supernatant) were passed through an amylose column with or without prebound MBP-Mep72 and eluted in 10 mM maltose. Lane 1, eluate from column precharged with MBP-Mep72 after adding 10 mM maltose; lane 2, eluate from control column after adding 10 mM maltose.

To further confirm the DiGE results, we used Western blot analysis to investigate whether PcrV and ExoS are differentially processed in the biofilm secretome of the *mep72* mutant compared with that in the wild type (Fig. 7A). Although ExoT may well be targeted by Mep72, since at least one spot (master no. 757) was unambiguously assigned as ExoS and the other spot (master no. 751) was assigned as either ExoS or ExoT (due to the high level of sequence similarity between the two proteins), we chose to focus our subsequent analyses on ExoS. ExoS displayed decreased apparent stability in the *mep72* mutant secretome, as evidenced by the appearance of multiple lower-molecular-weight bands. Unlike ExoS, which was found only in the culture supernatant, PcrV was identified by Western analyses in the culture supernatant and in the matrix surrounding the biofilm cells. In both of these fractions, PcrV migrated as a single band in the *mep72* mutant but with a lower apparent molecular weight. These data indicate that, contrary to expectation, Mep72 protects these targets against proteolytic processing/degradation.

### Mep72 and T3SS factors interact *in vitro*.

A pulldown experiment was conducted to determine if there was any physical interaction between Mep72 and the T3SS proteins. MBP-Mep72 was overexpressed in E. coli, refolded *in vitro*, and then immobilized on an amylose affinity matrix. Secreted proteins from a stationary-phase culture of PAO1 were allowed to bind, and the matrix was washed. Bound proteins were eluted, separated by SDS-PAGE, and probed by immunoblot analysis with antibodies raised against PcrV or ExoS (Fig. 7B). PcrV and ExoS coeluted with MBP-Mep72, suggesting a direct physical interaction between these proteins. As noted previously, since Mep72 was necessarily refolded *in vitro* for this experiment, these results are likely to underrepresent the interaction(s) occurring *in vivo*.

## DISCUSSION

In the current study, we used global proteomic analysis to directly compare the secretome of biofilms and planktonic cell cultures. Through this analysis, we identified a novel metzincin protease (Mep72) whose expression appears to be highly biofilm specific under these growth conditions. Further characterization of Mep72 revealed that it is secreted through the main terminal branch of the type II general secretory pathway (Xcp system) and is processed during (or possibly shortly after) export. We also found that in the absence of coexpressed *bamI*, heterologous *mep72* expression is toxic to E. coli, and that Mep72 and BamI form a protein-protein complex. *mep72* expression in biofilms was found to alter the electrophoretic mobility of a discrete subset of secreted proteins, especially those associated with T3S. Mep72 subsequently was shown to physically capture PcrV and ExoS *in vitro*.

Recently, Balyimez et al. presented a preliminary report showing that in wild-type PAO1, the PA2782-PA2783 genes are under the control of the CRP homologue Vfr (34). However, their studies were carried out using planktonic cultures, where PA2783 expression is, in our hands, very low. Consistent with this, when they examined the expression of PA2783 in the UWGC mutant bank strain, lacZbp02q4E12 (which encodes a *lacZ* fusion at position 28 of the ORF, the same PA2783 mutant that we used in the current study), Balyimez et al. found that β-galactosidase activity was negligible unless *vfr* levels were artificially elevated by expression from a high-copy-number plasmid (34). Similarly, alkaline phosphatase activity in cultures of PAO1 containing a plasmid-borne PA2783::*phoA* fusion also was extremely low. Balyimez et al. also found that when PA2783 is expressed in E. coli, the culture produces a zone of clearing when spotted onto skim-milk (protease) bioassay plates. However, we find this difficult to reconcile with the fact that E. coli lacks the main terminal branch of the general secretory pathway and lacks any mechanism by which to secrete proteases into the extracellular milieu. Instead, and given our results showing that the expression of PA2783 is toxic to E. coli, we suspect that the zone of clearing observed by these workers arose due to the release of intracellular proteases as a result of autolysis. Consistent with this, these workers failed to identify any secreted protease activity in concentrated supernatants from E. coli cultures overexpressing PA2783, although they did identify a low level of protease activity associated with the outer membrane fraction. Based on these findings, Balyimez et al. concluded that PA2783 is a membrane-bound 65-kDa endopeptidase and named it Mep72 (34). Our data suggest that Mep72 is in fact a truly secreted protein and that it exhibits minimal endopeptidase activity on generic substrates, such as milk protein. Furthermore, the protein undergoes autoprocessing during export to yield a ca. 25-kDa mature protein.

As with many other metzincins, we found that Mep72 undergoes extensive processing before yielding the mature extracellular form. In this regard, Mep72 shares similar genomic and biochemical properties with its sibling metzincin, AprA. Like AprA, Mep72 is coexpressed with an inhibitor protein, BamI, which presumably suppresses the toxic activity of the metzincin until the latter has been secreted (49, 50). However, unlike AprI (the inhibitor of AprA), which has a well-defined tertiary structure, BamI contains several regions that are predicted to be natively unfolded (data not shown). Moreover, although AprA has been shown to cleave generic protein substrates, such as gelatin and casein (52), we found no evidence that Mep72 is proteolytically active against these. The two metzincins also differ in their mode of export; while AprA has its own dedicated type I secretion system, Mep72 is secreted through the Xcp-encoded type II secretion pathway.

Only a small number of proteins in the *mep72* mutant secretome displayed altered mobility on 2D gels, but these included components of the T3S and motility apparatus. It has not escaped our notice that the T3S and flagellar apparatus are evolutionarily related and that the proteins which pass through these assemblages are among the few secreted proteins which bypass the periplasm completely during export. One interesting, albeit speculative, possibility is that as they pass through the periplasm, most P. aeruginosa secreted proteins become modified in some way that protects them from the action of potent (and in many cases, relatively nonspecific) cosecreted proteases, such as LasB. From an anthropocentric viewpoint, without such a mechanism, it may be counterproductive to secrete exoproteins into a preexisting, self-made proteolytic soup. Such protective posttranslational modifications may account for the “charge trains” commonly seen in 2D gels of P. aeruginosa secretome samples, a possibility that we are currently investigating. Presumably, given that the T3S and flagellar proteins bypass the periplasm, they do not receive any such protective modification. This may explain why these proteins in particular are targeted by Mep72, which, counterintuitively, appears to protect T3S proteins (PcrV and ExoS) from proteolysis (Fig. 7A). How Mep72 recognizes its target proteins is not known. Presumably, Mep72 recognizes specific sequence motifs in these targets. Alternatively, it may be that the absence of a protective periplasm-conferred modification (that is normally present on other secreted proteins) promotes Mep72 binding. Posttranslational modification of extracellular proteins is not in itself unusual; as noted by Nouwens and colleagues, a plethora of secreted proteins undergo posttranslational modification in P. aeruginosa, leading to the formation of multiple distinct spots of the same protein on 2D gels (27). Indeed, LasB is represented by as many as 11 discrete spots (or processed isoforms), and the LasA protease is known to be processed by other secreted endopeptidases, including LasB (53, 54). It is possible that Mep72 contributes to this complex network of processing by protecting its substrates from proteolysis by other secreted proteases.

Mep72 was shown to influence the electrophoretic mobility of a number of structural components of the T3SS translocation complex (PcrV and PopD). Here, we demonstrate a physical interaction between Mep72 and PcrV by an *in vitro* pulldown assay. However, it remains unclear whether Mep72 associates with assembled but surface-detached (broken) needle complexes or whether PcrV and PopD are truly secreted proteins. This is still an area of active research; for example, a study by Akopyan and colleagues demonstrated that translocators and effectors of the Yersinia T3SS localized to the surface of bacterial cells before target cell contact (55).

Purified Mep72 underwent rapid and essentially complete autodigestion following refolding *in vitro*. This feature may be physiologically relevant (e.g., serving to self-limit the accumulation of the protein in culture supernatants), or it may be an artifact of the refolding procedure; loosely folded or partially folded intermediates may be more susceptible to proteolysis by small amounts of correctly folded protease. One possible missing refolding factor was zinc ions. However, the addition of supplementary zinc to the refolding buffer had no impact on the autolytic kinetics of the refolded His_6_-tagged protein (data not shown). Another possible refolding factor is BamI. This protein clearly confers protection upon cells expressing Mep72 (Fig. 5A), although its precise mode of action is uncertain. It remains unclear whether this protein simply binds to Mep72 and inhibits its protease activity (as in the AprI-AprA interaction) or whether this binding simply prevents Mep72 from recognizing its target proteins. These are open questions that we are currently investigating further.

Curiously, a nucleotide substitution that led to the introduction of a stop codon 5′ of the carbohydrate-binding domains obviated toxicity, as revealed by a bypass screen. Additionally, expression of neither the metzincin domain nor carbohydrate-binding domains alone resulted in toxicity. This suggests that all 3 domains must be present for toxicity to be manifest. One possibility is that all three domains are required for proper folding of the protein.

In summary, we have demonstrated that the secretome of planktonic cell cultures is very different from that of biofilms. This work led to the discovery and subsequent characterization of Mep72, a novel metzincin. Current work is aimed at better understanding the precise mechanism by which Mep72 affects the T3S proteins and the biochemical consequences of this processing. We are also investigating what regulates *bamI-mep72* expression in biofilms.

## References

[1] Lam J, Chan R, Lam K, Costerton JW 1980 Production of mucoid microcolonies by Pseudomonas aeruginosa within infected lungs in cystic fibrosis. Infect Immun 28:546–556. 6772562 10.1128/iai.28.2.546-556.1980 PMC550970

[2] Govan JR, Deretic V 1996 Microbial pathogenesis in cystic fibrosis: mucoid Pseudomonas aeruginosa and Burkholderia cepacia. Microbiol Rev 60:539–574. 8840786 10.1128/mr.60.3.539-574.1996 PMC239456

[3] Costerton JW, Stewart PS, Greenberg EP 1999 Bacterial biofilms: a common cause of persistent infections. Science 284:1318–1322. doi:10.1126/science.284.5418.1318. 10334980

[4] Singh PK, Schaefer AL, Parsek MR, Moninger TO, Welsh MJ, Greenberg EP 2000 Quorum-sensing signals indicate that cystic fibrosis lungs are infected with bacterial biofilms. Nature 407:762–764. doi:10.1038/35037627. 11048725

[5] Lewis K 2001 Riddle of biofilm resistance. Antimicrob Agents Chemother 45:999–1007. doi:10.1128/AAC.45.4.999-1007.2001. 11257008 PMC90417

[6] Parsek MR, Singh PK 2003 Bacterial biofilms: an emerging link to disease pathogenesis. Annu Rev Microbiol 57:677–701. doi:10.1146/annurev.micro.57.030502.090720. 14527295

[7] Whiteley M, Bangera MG, Bumgarner RE, Parsek MR, Teitzel GM, Lory S, Greenberg EP 2001 Gene expression in Pseudomonas aeruginosa biofilms. Nature 413:860–864. doi:10.1038/35101627. 11677611

[8] Sauer K, Camper AK, Ehrlich GD, Costerton JW, Davies DG 2002 Pseudomonas aeruginosa displays multiple phenotypes during development as a biofilm. J Bacteriol 184:1140–1154. doi:10.1128/jb.184.4.1140-1154.2002. 11807075 PMC134825

[9] Hentzer M, Eberl L, Givskov M 2005 Transcriptome analysis of Pseudomonas aeruginosa biofilm development: anaerobic respiration and iron limitation. Biofilms 2:37–61. doi:10.1017/S1479050505001699.

[10] Waite RD, Papakonstantinopoulou A, Littler E, Curtis MA 2005 Transcriptome analysis of Pseudomonas aeruginosa growth: comparison of gene expression in planktonic cultures and developing and mature biofilms. J Bacteriol 187:6571–6576. doi:10.1128/JB.187.18.6571-6576.2005. 16159792 PMC1236618

[11] Mikkelsen H, Bond NJ, Skindersoe ME, Givskov M, Lilley KS, Welch M 2009 Biofilms and type III secretion are not mutually exclusive in Pseudomonas aeruginosa. Microbiology 155:687–698. doi:10.1099/mic.0.025551-0. 19246740

[12] Dötsch A, Eckweiler D, Schniederjans M, Zimmermann A, Jensen V, Scharfe M, Geffers R, Häussler S 2012 The Pseudomonas aeruginosa transcriptome in planktonic cultures and static biofilms using RNA sequencing. PLoS One 7:e31092. doi:10.1371/journal.pone.0031092. 22319605 PMC3272035

[13] Patell S, Gu M, Davenport P, Givskov M, Waite RD, Welch M 2010 Comparative microarray analysis reveals that the core biofilm-associated transcriptome of Pseudomonas aeruginosa comprises relatively few genes. Environ Microbiol Rep 2:440–448. doi:10.1111/j.1758-2229.2010.00158.x. 23766118

[14] Van Delden C, Iglewski BH 1998 Cell-to-cell signaling and Pseudomonas aeruginosa infections. Emerg Infect Dis 4:551–560. doi:10.3201/eid0404.980405. 9866731 PMC2640238

[15] Bleves S, Viarre V, Salacha R, Michel GPF, Filloux A, Voulhoux R 2010 Protein secretion systems in Pseudomonas aeruginosa: a wealth of pathogenic weapons. Int J Med Microbiol 300:534–543. doi:10.1016/j.ijmm.2010.08.005. 20947426

[16] Yahr TL, Goranson J, Frank DW 1996 Exoenzyme S of Pseudomonas aeruginosa is secreted by a type III pathway. Mol Microbiol 22:991–1003. doi:10.1046/j.1365-2958.1996.01554.x. 8971719

[17] Yahr TL, Mende-Mueller LM, Friese MB, Frank DW 1997 Identification of type III secreted products of the Pseudomonas aeruginosa exoenzyme S regulon. J Bacteriol 179:7165–7168. 9371466 10.1128/jb.179.22.7165-7168.1997 PMC179660

[18] Hauser AR, Cobb E, Bodi M, Mariscal D, Vallés J, Engel JN, Rello J 2002 Type III protein secretion is associated with poor clinical outcomes in patients with ventilator-associated pneumonia caused by Pseudomonas aeruginosa. Crit Care Med 30:521–528. doi:10.1097/00003246-200203000-00005. 11990909

[19] Roy-Burman A, Savel RH, Racine S, Swanson BL, Revadigar NS, Fujimoto J, Sawa T, Frank DW, Wiener-Kronish JP 2001 Type III protein secretion is associated with death in lower respiratory and systemic Pseudomonas aeruginosa infections. J Infect Dis 183:1767–1774. doi:10.1086/320737. 11372029

[20] Goodman AL, Kulasekara B, Rietsch A, Boyd D, Smith RS, Lory S 2004 A signaling network reciprocally regulates genes associated with acute infection and chronic persistence in Pseudomonas aeruginosa. Dev Cell 7:745–754. doi:10.1016/j.devcel.2004.08.020. 15525535

[21] Ventre I, Goodman AL, Vallet-Gely I, Vasseur P, Soscia C, Molin S, Bleves S, Lazdunski A, Lory S, Filloux A 2006 Multiple sensors control reciprocal expression of Pseudomonas aeruginosa regulatory RNA and virulence genes. Proc Natl Acad Sci U S A 103:171–176. doi:10.1073/pnas.0507407103. 16373506 PMC1324988

[22] Furukawa S, Kuchma SL, O'Toole GA 2006 Keeping their options open: acute versus persistent infections. J Bacteriol 188:1211–1217. doi:10.1128/JB.188.4.1211-1217.2006. 16452401 PMC1367219

[23] Schaber JA, Triffo WJ, Suh SJ, Oliver JW, Hastert MC, Griswold JA, Auer M, Hamood AN, Rumbaugh KP 2007 Pseudomonas aeruginosa forms biofilms in acute infection independent of cell-to-cell signaling. Infect Immun 75:3715–3721. doi:10.1128/IAI.00586-07. 17562773 PMC1952004

[24] Yang L, Haagensen JAJ, Jelsbak L, Johansen HK, Sternberg C, Høiby N, Molin S 2008 In situ growth rates and biofilm development of Pseudomonas aeruginosa populations in chronic lung infections. J Bacteriol 190:2767–2776. doi:10.1128/JB.01581-07. 18156255 PMC2293235

[25] Horsman SR, Moore RA, Lewenza S 2012 Calcium chelation by alginate activates the type III secretion system in mucoid Pseudomonas aeruginosa biofilms. PLoS One 7:e46826. doi:10.1371/journal.pone.0046826. 23056471 PMC3466208

[26] Arevalo-Ferro C, Hentzer M, Reil G, Görg A, Kjelleberg S, Givskov M, Riedel K, Eberl L 2003 Identification of quorum-sensing regulated proteins in the opportunistic pathogen Pseudomonas aeruginosa by proteomics. Environ Microbiol 5:1350–1369. doi:10.1046/j.1462-2920.2003.00532.x. 14641579

[27] Nouwens AS, Beatson SA, Whitchurch CB, Walsh BJ, Schweizer HP, Mattick JS, Cordwell SJ 2003 Proteome analysis of extracellular proteins regulated by the las and rhl quorum sensing systems in Pseudomonas aeruginosa PAO1. Microbiology 149:1311–1322. doi:10.1099/mic.0.25967-0. 12724392

[28] Kim E-J, Wang W, Deckwer W-D, Zeng A-P 2005 Expression of the quorum-sensing regulatory protein LasR is strongly affected by iron and oxygen concentrations in cultures of Pseudomonas aeruginosa irrespective of cell density. Microbiology 151:1127–1138. doi:10.1099/mic.0.27566-0. 15817780

[29] Nalca Y, Jänsch L, Bredenbruch F, Geffers R, Buer J, Häussler S 2006 Quorum-sensing antagonistic activities of azithromycin in Pseudomonas aeruginosa PAO1: a global approach. Antimicrob Agents Chemother 50:1680–1688. doi:10.1128/AAC.50.5.1680-1688.2006. 16641435 PMC1472232

[30] Toyofuku M, Roschitzki B, Riedel K, Eberl L 2012 Identification of proteins associated with the Pseudomonas aeruginosa biofilm extracellular matrix. J Proteome Res 11:4906–4915. doi:10.1021/pr300395j. 22909304

[31] Sarkisova S, Patrauchan MA, Berglund D, Nivens DE, Franklin MJ 2005 Calcium-induced virulence factors associated with the extracellular matrix of mucoid Pseudomonas aeruginosa biofilms. J Bacteriol 187:4327–4337. doi:10.1128/JB.187.13.4327-4337.2005. 15968041 PMC1151780

[32] Purschke FG, Hiller E, Trick I, Rupp S 2012 Flexible survival strategies of Pseudomonas aeruginosa in biofilms result in increased fitness compared with Candida albicans. Mol Cell Proteomics 11:1652–1669. doi:10.1074/mcp.M112.017673. 22942357 PMC3518115

[33] Borlee BR, Goldman AD, Murakami K, Samudrala R, Wozniak DJ, Parsek MR 2010 Pseudomonas aeruginosa uses a cyclic-di-GMP-regulated adhesin to reinforce the biofilm extracellular matrix. Mol Microbiol 75:827–842. doi:10.1111/j.1365-2958.2009.06991.x. 20088866 PMC2847200

[34] Balyimez A, Colmer-Hamood JA, Francisco MS, Hamood AN 2013 Characterization of the Pseudomonas aeruginosa metalloendopeptidase, Mep72, a member of the Vfr regulon. BMC Microbiol 13:269. doi:10.1186/1471-2180-13-269. 24279383 PMC4222646

[35] Mikkelsen H, Duck Z, Lilley KS, Welch M 2007 Interrelationships between colonies, biofilms, and planktonic cells of Pseudomonas aeruginosa. J Bacteriol 189:2411–2416. doi:10.1128/JB.01687-06. 17220232 PMC1899361

[36] Kwon DH, Lu C-D 2006 Polyamines induce resistance to cationic peptide, aminoglycoside, and quinolone antibiotics in Pseudomonas aeruginosa PAO1. Antimicrob Agents Chemother 50:1615–1622. doi:10.1128/AAC.50.5.1615-1622.2006. 16641426 PMC1472189

[37] Kwon DH, Lu C-D 2006 Polyamines increase antibiotic susceptibility in Pseudomonas aeruginosa. Antimicrob Agents Chemother 50:1623–1627. doi:10.1128/AAC.50.5.1623-1627.2006. 16641427 PMC1472196

[38] Kwon D-H, Lu C-D 2007 Polyamine effects on antibiotic susceptibility in bacteria. Antimicrob Agents Chemother 51:2070–2077. doi:10.1128/AAC.01472-06. 17438056 PMC1891406

[39] Wilderman PJ, Vasil AI, Johnson Z, Wilson MJ, Cunliffe HE, Lamont IL, Vasil ML 2001 Characterization of an endoprotease (PrpL) encoded by a PvdS-regulated gene in Pseudomonas aeruginosa. Infect Immun 69:5385–5394. doi:10.1128/IAI.69.9.5385-5394.2001. 11500408 PMC98648

[40] Folders J, Tommassen J, van Loon LC, Bitter W 2000 Identification of a chitin-binding protein secreted by Pseudomonas aeruginosa. J Bacteriol 182:1257–1263. doi:10.1128/JB.182.5.1257-1263.2000. 10671445 PMC94410

[41] Mao F, Dam P, Chou J, Olman V, Xu Y 2009 DOOR: a database for prokaryotic operons. Nucleic Acids Res 37:D459–D463. doi:10.1093/nar/gkn757. 18988623 PMC2686520

[42] Murzin AG 1992 Structural principles for the propeller assembly of beta-sheets: the preference for seven-fold symmetry. Proteins 14:191–201. doi:10.1002/prot.340140206. 1409568

[43] Yeats C, Bentley S, Bateman A 2003 New knowledge from old: in silico discovery of novel protein domains in Streptomyces coelicolor. BMC Microbiol 3:3. doi:10.1186/1471-2180-3-3. 12625841 PMC151604

[44] Shi J, Blundell TL, Mizuguchi K 2001 FUGUE: sequence-structure homology recognition using environment-specific substitution tables and structure-dependent gap penalties. J Mol Biol 310:243–257. doi:10.1006/jmbi.2001.4762. 11419950

[45] Bode W, Grams F, Reinemer P, Gomis-Rüth FX, Baumann U, McKay DB, Stöcker W 1996 The metzincin-superfamily of zinc-peptidases. Adv Exp Med Biol 389:1–11. doi:10.1007/978-1-4613-0335-0_1. 8860988

[46] Gomis-Rüth FX 2003 Structural aspects of the metzincin clan of metalloendopeptidases. Mol Biotechnol 24:157–202. doi:10.1385/MB:24:2:157. 12746556

[47] Gilkes NR, Henrissat B, Kilburn DG, Miller RC, Warren RA 1991 Domains in microbial beta-1,4-glycanases: sequence conservation, function, and enzyme families. Microbiol Rev 55:303–315. 1886523 10.1128/mr.55.2.303-315.1991 PMC372816

[48] Ball G, Chapon-Hervé V, Bleves S, Michel G, Bally M 1999 Assembly of XcpR in the cytoplasmic membrane is required for extracellular protein secretion in Pseudomonas aeruginosa. J Bacteriol 181:382–388. 9882649 10.1128/jb.181.2.382-388.1999 PMC93389

[49] Feltzer RE, Gray RD, Dean WL, Pierce WM 2000 Alkaline proteinase inhibitor of Pseudomonas aeruginosa. Interaction of native and N-terminally truncated inhibitor proteins with Pseudomonas metalloproteinases. J Biol Chem 275:21002–21009. doi:10.1074/jbc.M002088200. 10770939

[50] Hege T, Feltzer RE, Gray RD, Baumann U 2001 Crystal structure of a complex between Pseudomonas aeruginosa alkaline protease and its cognate inhibitor: inhibition by a zinc-NH2 coordinative bond. J Biol Chem 276:35087–35092. doi:10.1074/jbc.M104020200. 11445573

[51] Bardoel BW, van Kessel KPM, van Strijp JAG, Milder FJ 2012 Inhibition of Pseudomonas aeruginosa virulence: characterization of the AprA-AprI interface and species selectivity. J Mol Biol 415:573–583. doi:10.1016/j.jmb.2011.11.039. 22154939

[52] Caballero AR, Moreau JM, Engel LS, Marquart ME, Hill JM, O'Callaghan RJ 2001 Pseudomonas aeruginosa protease IV enzyme assays and comparison to other Pseudomonas proteases. Anal Biochem 290:330–337. doi:10.1006/abio.2001.4999. 11237336

[53] Kessler E, Safrin M, Gustin JK, Ohman DE 1998 Elastase and the LasA protease of Pseudomonas aeruginosa are secreted with their propeptides. J Biol Chem 273:30225–30231. doi:10.1074/jbc.273.46.30225. 9804780

[54] Braun P, de Groot A, Bitter W, Tommassen J 1998 Secretion of elastinolytic enzymes and their propeptides by Pseudomonas aeruginosa. J Bacteriol 180:3467–3469. 9642203 10.1128/jb.180.13.3467-3469.1998 PMC107305

[55] Akopyan K, Edgren T, Wang-Edgren H, Rosqvist R, Fahlgren A, Wolf-Watz H, Fallman M 2011 Translocation of surface-localized effectors in type III secretion. Proc Natl Acad Sci U S A 108:1639–1644. doi:10.1073/pnas.1013888108. 21220342 PMC3029700

